# Peer support through video meetings: Experiences of people with young onset dementia

**DOI:** 10.1177/14713012221140468

**Published:** 2022-11-18

**Authors:** Esther Vera Gerritzen, Gianna Kohl, Martin Orrell, Orii McDermott

**Affiliations:** Institute of Mental Health, Mental Health and Clinical Neuroscience, School of Medicine, 6123 University of Nottingham, Nottingham, UK; Research Department of Clinical, Educational and Health Psychology, 4919 University College London, London, UK; Institute of Mental Health, Mental Health and Clinical Neuroscience, School of Medicine, 6123 University of Nottingham, Nottingham, UK

**Keywords:** young onset dementia, peer support, online, video meetings, focus groups

## Abstract

**Background:**

People with young onset dementia can experience stigmatization and social isolation. Peer support provides an opportunity for social connection and support. However, access to in-person peer support groups varies across the UK, and during the COVID-19 pandemic in-person peer support groups moved online.

**Objectives:**

We explored the experiences of people with young onset dementia attending peer support meetings through online videoconferencing platforms, and identified barriers and facilitators.

**Methods:**

A focus group study with existing peer support groups for people with young onset dementia that had their meetings online was conducted. Participants were people living with young onset dementia. Participants were recruited through convenience and purposeful sampling. Initial contact was made with the group facilitator to discuss the study purposes and assess eligibility of the group members. The data was analysed thematically by two independent researchers, using an inductive approach.

**Findings:**

Four focus groups with UK-based peer support groups were conducted through the group’s usual platform, including 20 participants. Through online peer support people stayed connected during the pandemic. It provided opportunities to meet people from different places and be involved in research projects. People found it a convenient way of connecting with others without having to travel. However, some missed in-person interaction and digital exclusion and challenges to navigate different platforms were identified as barriers. Organisational skills of the facilitator and support with getting into meetings can help overcome these challenges.

**Conclusion:**

Online platforms can make peer support more accessible for people with young onset dementia as it overcomes geographical barriers as well as barriers for those who feel uncomfortable attending an in-person group. Researchers and policy makers should explore how to implement and overcome barriers to online peer support, so that peer support is more widely accessible and clearly signposted to people with young onset dementia.

## Introduction

### Background

Dementia is mostly prevalent in the older population, however, it also affects younger people. People with young onset dementia are diagnosed before the age of 65 (Koopmans & Rosness, 2014). They are often in a different phase in life than older adults and have different needs and face different challenges (Millenaar et al., 2017; Oliver et al., 2019). People with young onset dementia are more likely to still be in employment, fulfil an active parenting role towards dependent children, or have responsibilities towards older parents (Roach, 2016). Receiving a dementia diagnosis at such a young age has a significant social and emotional impact (Rabanal et al., 2018). People with young onset dementia often experience a decrease in their social contacts and report losing touch with friends, which can be the result of a lack of understanding of young onset dementia in the wider society. More specifically, as dementia is commonly associated with older age, younger people with dementia often experience stigma (Bannon et al., 2020). Feelings of stigmatization can result in avoidance of social situations, which increases the risk of social isolation and loneliness (Johannessen et al., 2018).

One way to improve social connection and support is through peer support (Pierse et al., 2021). Peer support can be defined as the exchange of support between those (also referred to as ‘peers’) who share a similar health condition or life experience (Keyes et al., 2014; Solomon, 2004). Peer support is characterised by reciprocity of support, as peers can develop a relationship in which they can both receive and provide support (Barak et al., 2008; Keyes et al., 2014). Furthermore, peers can share experiential knowledge, which is information and perspectives that they have because of their own unique experiences of living with young onset dementia (Dennis, 2003). The reviews of Dugmore et al. (2015) and McDermott et al. (2019) highlight the importance of including peer support in psychosocial interventions, and Greenwood and Smith (2016) suggest that peer support can be beneficial and enjoyable for people with young onset dementia. Furthermore, Rabanal et al. (2018) found that peer support is a crucial source of post-diagnostic support for people living with young onset dementia. These findings are supported by Stamou, Fontaine, et al. (2021) who indicate that peer support can contribute to a more positive post-diagnostic experience and help people with young onset dementia and their families to identify support services.

Peer support can be provided in different ways, including the online setting. Examples include peer support through social media, discussion forums, and blogs. One of the main advantages of peer support in an online setting is that it overcomes geographical barriers (Moorhead et al., 2013). Access to specialised young onset dementia support services varies widely across the UK (Stamou, La Fontaine, et al., 2021), indicating that online peer support could be particularly helpful for those who have no access to specialised (peer) support services in their local area, or who are unable to travel.

Research into how people with dementia use social media and online support is increasing. Clare et al. (2008) and Rodriquez (2013) show that people with dementia use discussion forums to exchange support and be part of a community. Craig and Strivens (2016) analysed an open Facebook group for people with young onset dementia and their supporters in which users expressed themselves, provided support, and raised awareness. However, it remains unclear how well it supported the needs of people with young onset dementia (Craig & Strivens, 2016). Finally, Talbot et al. (2020) found that Twitter was used by people with dementia for collective action, to represent their needs and experiences, to create awareness, and to a lesser extent for social support. While these studies show how people with (young onset) dementia use online platforms for peer support, it remains unknown how users actually experience this and how it impacts their daily lives.

This work is part of a larger study which aims to develop best practice guidance on online peer support for people with young onset dementia. This study will use a variety of methods to gain insights into how people with young onset dementia use and experience different forms of online peer support, and what the potential barriers are (Gerritzen et al., 2022). Until now, research on online peer support for people with dementia focusses on text-based platforms. During the COVID-19 pandemic and national lockdowns, videoconferencing platforms such as Zoom, Skype, and MS Teams became more popular. During this time when many in-person (peer) support services for people with dementia were disrupted, some adapted to online platforms (Giebel et al., 2021). Research has been conducted into using videoconferencing platforms for (peer) support for informal carers of people with dementia (Banbury et al., 2019), yet, no research has been conducted into how people with young onset dementia experience using videoconferencing platforms for peer support.

### Objectives

This study aims to (1) explore how people with young onset dementia experience peer support through videoconferencing platforms, and (2) identify barriers and facilitators of peer support through videoconferencing platforms.

## Methods

This study received ethical approval from the London Bromley Research Ethics Committee (reference number: 21/LO/0248). For this qualitative study, focus groups were conducted to explore how people with young onset dementia experience participating in an online peer support group through videoconferencing platforms. The focus groups were held with existing peer support groups, which were all based in the UK. The results are reported following the consolidated criteria for reporting qualitative research (COREQ) (Tong et al., 2007).

### Recruitment

In May and June 2021 existing peer support groups were recruited using a combination of convenience and purposeful sampling. For the convenience sampling method, the study was advertised through (1) an online Patient and Public Involvement event, and (2) the Dementia Engagement and Empowerment Project (DEEP), which is a UK-based network of support groups for people with dementia. People who were interested in taking part could contact the first author (EVG). For the purposeful sampling method, EVG contacted peer support facilitators in her professional network. This included facilitators from (1) the Young Dementia Network, which is a collaborative network consisting of people living with young onset dementia, their supporters, and professionals, and (2) the Rare Dementia Support (RDS) service.

Initial contact with the peer support group was made through the group facilitator. Before each focus group EVG had a meeting with the group facilitator to learn a bit more about the group, explain the purpose of the study, and assess the eligibility of the group members. Next, the facilitator shared the study with the group and gathered interest among the members. EVG also offered to present the study during one of the group’s meetings. This was done for two of the four focus groups. In both instances no issues or concerns were raised, and members expressed interest in taking part in the focus group.

### Eligibility criteria

People were eligible for the study if (1) they were living with a dementia diagnosis, (2) they had received their diagnosis before the age of 65, and (3) they were part of an existing peer support group that met online. People did not have to be younger than 65 at the time they took part in the study, as long as they had received their diagnosis before the age of 65. In case that the support group included both people living with dementia and family members, EVG and the facilitator came to an agreement that, while the emphasis of the focus groups would be on the experiences of those living with dementia, family members could join if they wanted to. This was to not exclude some of the group members.

### Consent procedures

Each participant received a Participant Information Sheet and an Informed Consent form via email or per post, depending on their preference. Due to the COVID-19 pandemic and UK-wide lockdowns as well as the wide variety in geographical locations of the participants, informed consent was taken remotely. The informed consent process was offered in different formats to accommodate different needs and preferences of the participants. Participants could provide written or verbal consent. Written consent could be done by signing the consent form on paper or digitally and sending it back to EVG. For verbal consent EVG went through the study information and consent form over a videocall on MS Teams or over the phone, which was recorded (after the participant gave permission). All options were presented to the group facilitator, who would advise on the most suitable option for each participant.

### Focus group procedures

The focus groups were conducted online through the group’s usual meeting platform at a time and day that was convenient for the group. The aim was to organize 4–6 focus groups, as data saturation tends to occur after 4–6 focus groups have been conducted (Hennink et al., 2019). Each focus group was facilitated by EVG, who has a background in health sciences. A co-facilitator (GK) with a background in psychology was present to take field notes, monitor the chat, and ask additional questions. Both facilitators were early-career researchers. The group’s usual facilitator was present at the beginning of the meeting to welcome everyone, but was not there during the focus group itself. This was to ensure that people could speak freely about their experiences. EVG discussed this with the facilitators beforehand, who all agreed.

The focus groups were semi-structured using a pre-defined topic guide. The topic guide was informed by informal consultations with people with young onset dementia and professionals working with people with young onset dementia, literature research, and discussions within the research team. The informal consultations were held before developing the topic guide, to get an understanding of the challenges that people with young onset dementia face, in particular with finding peer support and using technology. The informal consultations did not bring up anything that was out of line with the literature. The topic guide covered (1) finding a peer support group, (2) general peer support experiences, (3) online peer support experiences and use of technology, and (4) hints and tips on coping with young onset dementia, finding support, and providing information and support for people with young onset dementia. The reason why the first two items were included in the topic guide is that some of the barriers of accessing a peer support group can be due to a lack of in-person services, stigma, and negative experiences with dementia and peer support services. The step towards online peer support might then be smaller compared to in-person groups.

### Data collection

The focus groups were screen- and audio-recorded using the recording function of the videoconferencing platform and an external recorder. The recordings were saved on EVG’s computer. Immediately afterwards the recording was uploaded onto a password secured online storage space of the University of Nottingham and deleted from the computer. The field notes were also saved on the password secured online storage space of the University of Nottingham. All focus groups were transcribed verbatim by Dictate2Us.

### Data analysis

The data was analysed through thematic analysis with an inductive approach using the procedures outlined by Braun and Clarke (2021). This consisted of six phases: (1) familiarising with the data, (2) coding the data, (3) developing initial themes, (4) developing and reviewing themes, (5) refining, defining and naming the themes, and (6) writing up.

#### Phase 1 and 2: familiarising with and coding the data

During the first phase, EVG and GK independently read the transcripts multiple times and wrote down and discussed their insights. Things that stood out for both authors were the difficulties during the post-diagnostic period and the losses that people faced, the positives of peer support in general, and missing not being together in person. For the second phase one transcript was selected to look at in more detail. Due to the amount of data it was decided to select a sample rather than using the full dataset. EVG, GK, and OM (a senior member of the research team with a background in music therapy), familiarised themselves with this transcript. During this phase the initial thoughts and ideas were refined and each author identified codes. In this context codes can be described as specific and detailed segments of the transcript that are potentially interesting and relevant (Braun & Clarke, 2021). This process was followed by a discussion between EVG, GK, and OM.

#### Phase 3, 4 and 5: developing and reviewing themes and writing up

During the third phase, EVG and GK went back to the selected transcript and generated initial themes. Themes describe a broader meaning rather than something very specific, as is the case for codes (Braun & Clarke, 2021). The initial themes were discussed among EVG and GK who developed an initial coding framework. Some examples of the initial themes are ‘time right after diagnosis’, ‘facilitators to joining a peer support group’, and ‘negatives of peer support in an online platform’. For the fourth phase the initial coding framework was applied to the selected transcript. EVG and GK independently coded the transcript to see whether the initial coding framework captured the important elements of the data and whether it showed relationships between the different themes. After discussing this process, EVG and GK refined the coding framework during the fifth phase. The main reason that refinement was needed was that there were too many themes and that at times it was difficult to know where a certain section of the data would fit best. During this phase the refined coding framework was applied to all transcripts and EVG and GK independently coded each transcript. Afterwards EVG and GK compared and discussed the results of the coding. Finally, during the sixth phase EVG took the lead in writing the manuscript, and the other authors provided detailed feedback.

### Trustworthiness of data

Triangulation was applied to ensure trustworthiness of the data. Multiple methods of data collection were used to achieve method triangulation. These included audio and screen recordings of the focus groups and field notes (Carter et al., 2014). The audio of what was spoken matched the body language observed in the screen recordings. The field notes gave an insight into which particular topics were important during each focus group, which was helpful during the first phase of the analysis process. Furthermore, the research team consisted of researchers in different stages of their career and with different professional backgrounds, ensuring investigator triangulation (Carter et al., 2014). Finally, member checking was used to ensure the analysis accurately reflected the participants’ experiences. The initial findings of the study were written up in a report and shared with the participants (Braun & Clarke, 2021). Nine participants expressed interest in receiving the initial findings of the study, of whom four provided feedback. All four agreed with the provisional findings, so there was no need to make changes.

### Participants

#### The groups

Four online focus groups were conducted. Three groups were mixed groups for people living with young onset dementia and family members and one was for people with a diagnosis only. Three groups were facilitated by a (healthcare) professional, whereas one was facilitated by a former family carer of someone with dementia. At the time of the focus groups, all groups were meeting once a month through a videoconferencing platform. Two groups existed before the COVID-19 pandemic and used to meet in person before lockdown measures came into place. Two groups were formed during lockdown, one with the intention to move to in-person meetings, and one was founded as an online-only group with the intention to include people from a wide geographical range.

Every participant took part from a place of their preference. Three focus groups were held on Zoom and one on GoToMeeting. The focus groups lasted between 73 and 120 min. One focus group lasted longer than the others because of technical problems and a longer break. In total 23 people expressed interest in taking part, including two family members. The family members were part of the group and were also there to supported the person with dementia to attend the meeting. Three people, including both family members, dropped out after signing the Informed Consent form. All three were not present at the start of their scheduled focus group. The group facilitator tried to contact them, but it is unknown to the research team why those members did not attend the focus group. This resulted in 20 people with young onset dementia taking part in the study. Of these, one person was based outside the UK. An overview of the focus groups is presented in Table 1.

**Table 1.** Focus group characteristics.

Group	Participants	Drop-outs	Platform used	Location and membership
1	2	1	Zoom	North England (local and regional members)
2	4	2	Zoom	Scotland (local and regional members)
3	6	0	GoToMeeting	South England (UK and international members)
4	8	0	Zoom	Central England (local and regional members)

#### Individual participants.

All participants were members of the support group. In one focus group, a participant joined the group for the first time, with the intention of becoming a member. Of the 20 participants, 11 were male and 9 were female, aged between 48 and 68. One participant received their formal diagnosis at the age of 68, however, the dementia symptoms started years before that. The most prevalent type of dementia among the participants was Alzheimer’s disease, followed by frontotemporal dementia. More information on the participant characteristics is presented in Table 2.

**Table 2.** Participant characteristics.

Variable	N
Gender	
Male (%)	11 (55%)
Female (%)	9 (45%)
Age	
Mean (min-max)	59.6 (48–68)
Dementia diagnosis	
Alzheimer’s disease (%)	7 (35%)
Frontotemporal dementia (%)	4 (20%)
Posterior cortical atrophy (%)	3 (15%)
Primary progressive aphasia (%)	3 (15%)
Lewy body dementia (%)	2 (10%)
Semantic dementia (%)	1 (5%)
Time since diagnosis	
< 1 year (%)	7 (35%)
1–2 years (%)	2 (10%)
2–3 years (%)	4 (20%)
> 3 years (%)	6 (30%)
Unknown (%)	1 (5%)
Living situation	
Living with partner (%)	9 (45%)
Living with partner and other family members (e.g. children) (%)	6 (30%)
Living alone (%)	3 (15%)
Other (%)	2 (10%)
Paid employment	
Yes (%)	4 (20%)
No (%)	16 (80%)

### Findings

**Figure 1.**
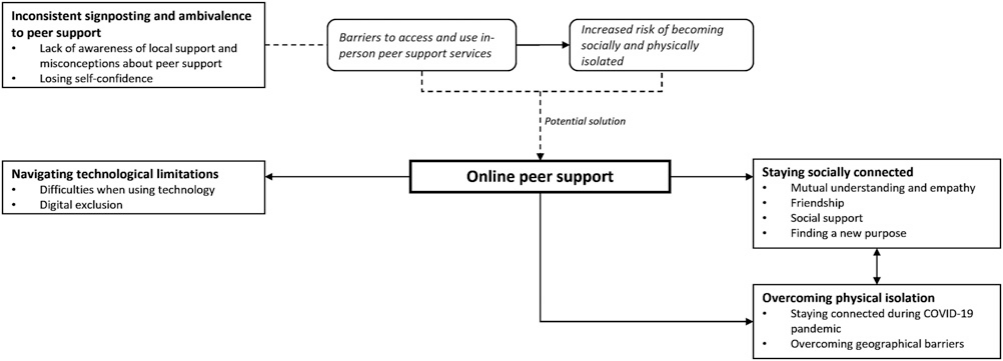
Overarching themes and subthemes.

Four overarching themes including nine subthemes were identified (Table 3 and Figure 1).

**Table 3.** Overarching themes and subthemes.

Overarching theme	Subthemes
1. Inconsistent signposting and ambivalence to peer support	• Lack of awareness of local support and misconceptions about peer support
	• Losing self-confidence
2. Staying socially connected	• Mutual understanding and empathy
	• Friendship and social support
	• Finding a new purpose
3. Overcoming physical isolation	• Staying connected during COVID-19 pandemic
	• Overcoming geographical barriers
4. Navigating technological limitations	• Difficulties when using technology
	• Digital exclusion

#### Theme 1: Inconsistent signposting and ambivalence to peer support

People frequently spoke about the challenges they faced in identifying (age-appropriate) support services and the lack of signposting. Across the groups there was a variety in available support groups and resources, with some areas having excellent services, and some having hardly any at all, or support was difficult to find.

*People promise you things and then sometimes they don’t come through with the promise […] It’s unfortunate we have to be so proactive, particularly given the fact that sometimes we find it hard to be proactive and we wish people would come and knock on our door and say ‘Look. Hello. Come and play with us today’*. (P10, group 3)

##### Lack of awareness of local support and misconceptions about peer support

People mentioned that before they joined their group, they were often unaware that there were groups specifically for younger people. This relates to some of the misconceptions that exist around peer support. For example, some people felt that peer support groups were only for older people and were about ‘singing songs about the war’. Some had previous negative experiences with support groups that were not age-appropriate, or experiences with an older parent with dementia.

*There was a lot of trepidation because I didn’t realise that there was a group that was very similar age, similar diagnosis etcetera […] I expected it would just be a lot of old people in their 70s and 80s*. (P1, group 1)

*I didn’t want to join a group because my mum’s experience was going away in a mini bus and disappearing for the day […] I had no idea what she did, I had no idea how she got on other than what she told me*. (P5, group 2)

##### Losing self-confidence

Receiving the diagnosis was a life-changing experience and was accompanied by uncertainty about the future and where to find support. People also experienced many losses, such as losing their jobs, having to give up driving, and losing contact with friends. For many, these losses resulted in losing their self-confidence as well.

*As soon as I was made to give up my job it felt like all the doors that were open to me before had shut […] People say ‘Oh, you know, you’d better not do that and you can’t do this. It’s unrealistic’. Expectations of yourself and from other people go right down. (P7, group 3)*

*[…] you lose your independence (P5, group 2), you lose your self-worth (P6, group 2)… The thought of joining a group of people when really, all you’ve had is your confidence kicked down, it’s really pretty hard*. (P5, group 2)

#### Theme 2: Staying socially connected

While some people said they had doubts before joining a group, they all agreed that joining the group was the right decision for them because it had a positive impact on their lives. People who participated in this study recommend to those who are reluctant to join a peer support group to just give it a try. They highlighted that there is a wide variety of topics and activities to take part in, that there is no need to say or do anything if you do not want to, and that there is no pressure to join every single meeting.

*I was silent for quite a long time when we started because A. I didn’t know what to say, and B. I didn’t really want to be there, [I was] kind of in denial with everything. But gradually I thought ‘actually this is alright’. It’s like with any sort of introduction to anybody, it takes a little while to get in there, but it’s definitely worth it*. (P14, group 4)

##### Mutual understanding and empathy

One of the most important aspects of peer support was the mutual understanding and acceptance within the group. “There’s lots of things we do not have to say to each other because we live in the same fog” (P4, group 2).

*You feel like you are losing yourself and you have things that you can’t necessarily discuss with other people, or feel awkward or uncomfortable about. But here you don’t feel uncomfortable because people understand. You are on the same level*. (P7, group 3)

People explained that others, whether it is family members, friends, or healthcare professionals, do not always understand what it is like to have young onset dementia.

*You might have your partner living with you, but you still feel alone to a certain extent because your partner cannot understand what you’re going through*. (P1, group 1)

##### Friendship and social support

People shared that their group was a great source of friendship and support and that it helped them to manage daily life with dementia. Through peer support people could share hints and tips for the challenges they face in their daily lives.

*We have had the privilege of being in each other’s company, meeting lifelong friendships, and that’s what gets you through the days and the months and hopefully the years […] it’s much more than that, it’s about enjoying them and making the most of them*. (P5, group 2)

*When you hear what people do with their lives, how they go about everyday living with this diagnosis, that they can achieve lots of things, it gives each one of us hope that you can keep going and do lots of things.* (P7, group 3)

##### Finding a new purpose

Peer support also offered people new opportunities for meaningful activities, involving research and advocacy, but also creative and arts-based activities that people might not have considered if it were not for the support group. The same is true for the opportunity to meet new people.

*We are going to have a dance group going on. I have a Zoom dance for different groups in [location] and we are going to start one for this group*. (P9, group 3)

*We’ve done so many things, we’ve been part of groups that have created training schedules […] Zoom has given us a purpose […] You think ‘I’ve made a difference. I’ve helped, I did something that’s really worthwhile’*. (P5, group 2)

People mentioned that a structure and an agenda for the meeting with specific topics can be helpful, but it can also be nice to have no structure and to use the meeting to just socialise and have a good time. Whether to have an agenda or not and what to discuss during the meeting was mostly a collaboration between the facilitator and the group. Moreover, people emphasized that the group was there for them, to meet their needs and wishes. The role of the facilitator was mainly described as providing the platform and connecting people, and to not talk too much. One group had two different meetings. One was reserved for more serious and dementia-related topics, and one was more of a social meeting. About the social meeting someone said:

*It is kind of a break from dementia. We focus on our interests, our opinions, how things are going for us in our personal lives, it’s a very rich experience […]. It’s an open slot for us to contribute in any way that we want to share.* (P20, group 4)

#### Theme 3: Overcoming physical isolation

Online peer support can overcome some of the limitations of in-person peer support. Not everyone may be able or feel comfortable to attend an in-person peer support group. Meeting a new group of people and speaking openly about one’s experiences with dementia can be daunting. One of the main positives of online peer support that people spoke about is being able to join from the comfort of their own home.

*You can feel a bit more comfortable when you are in your own home […] I’ve got my dogs here and I’m quite comfortable*. (P12, group 3)

*For those of us who are nervous about public speaking, Zoom is actually better because you are in your own environment. You can mute. You can fiddle about on the desk or whatever. You don’t get told off either. It has got its advantages*. (P10, group 3)

Furthermore, depending on the type of online setting, support could be readily available when needed. One group also had a WhatsApp group. Here people shared information and things that were going on in their lives, but it was also a place for people to share when they were feeling low. There would always be someone from the group to talk to. Furthermore, someone said the following about the WhatsApp group:

*You don’t feel obliged that you’ve got to respond. It just feels as if you are still in touch, that you’re not in your own little silo because other things are going on and it’s great to see what other people are doing*. (P8, group 3)

##### Staying connected during COVID-19 pandemic

Videoconferencing platforms allowed people to stay connected during the COVID-19 pandemic. People shared how important their peer support group was during this difficult time.

*I felt obviously on my own [but] I know that they are there at the other end of the computer and I just feel like I was part in their day*. (P3, group 2)

*I think if we didn’t have Zoom, I don’t know how I would be, but having the support in the group, even though it’s on Zoom, it is support and you can talk to people who are in the same shoes*. (P15, group 4)

##### Overcoming geographical barriers

Online meetings also allowed people to connect with others from different places, and provided opportunities to get involved in, for example, research projects across the country. Furthermore, it was a convenient way to stay connected.

*I’ve had some struggle to get out. Even if it was a local meeting I’d possibly have had problems actually physically getting there. Being able to have this, it breaks down lots of barriers because of distance, we can meet wherever we are internationally, but also if you struggle mobility wise or with anxiety about getting places, that sort of thing, it takes that away*. (P7, group 3)

#### Theme 4: Navigating technological limitations

Online peer support and technology in general has its limitations. People frequently mentioned that online meetings cannot replace in-person interaction and that this was something they missed.

*Whilst Zoom meetings are well and good, they’re not the same as meeting people and having cups of tea and eating biscuits and just doing that human connection thing that’s so important*. (P4, group 2)

##### Difficulties using technology

People shared that they sometimes had difficulties with joining a video meeting and navigating different platforms could be confusing.

*I was used to the Zoom platform and where all the buttons were […] but coming on this sometimes that’s a bit of a challenge because things are in different places. […] It can be very distracting*. (P8, group 3)

To overcome such challenges, people would message or call each other, and someone else would help them find the link or get in the meeting. Members of the group and their families also helped each other with setting up the technology, and for example installing Zoom. Facilitators can also play an important role in this. People agreed that it is important that the facilitator has good organisational skills to overcome these challenges, for example, by sending out timely reminders and providing clear instructions on how to join the meeting.

##### Digital exclusion

While all participants in this study were successfully using technology and videoconferencing platforms, digital exclusion was mentioned as an important limitation. Some of the groups used to meet in person before the COVID-19 pandemic. They noticed that some members of their group did not manage to take part in the online meetings, and therefore lost contact with the group. People felt that the main reason why other group members were unable to take part was that their dementia symptoms made it too difficult.

*The sad bit is that several members of the group can’t access Zoom […]. I really feel a loss for some of the folk who just not have had the same connection or same continuity that some of us have had and that’s quite sad because they’re very important members of the group*. (P5, group 2)

## Discussion

### Key findings

This study echoes findings of previous research (Clare et al., 2008; Craig & Strivens, 2016; Rodriquez, 2013; Talbot et al., 2020) showing that beneficial elements of peer support, such as emotional and social support, friendship building, and sharing experiences and information (Keyes et al., 2014), are not limited to in-person settings. Moreover, this study adds to previous research that the benefits of online peer support can go beyond text-based platforms, and are also present in video meetings. During the COVID-19 pandemic, for some the video meetings were better than having no support at all. However, this study also shows that peer support through video meetings is more than just a replacement of in-person peer support in times of worldwide disruption and crisis, and has its own unique benefits.

#### Benefits of online peer support using videoconferencing platforms

Peer support can be extremely beneficial for people with young onset dementia as it can make the post-diagnostic experience more positive (Rabanal et al., 2018; Stamou, Fontaine, et al., 2021) and reduce the risk of social isolation (Pierse et al., 2021). These findings suggest that every person with young onset dementia should have access to peer support. However, people often experience difficulties in accessing age-appropriate, local (peer) support services (Cations et al., 2017; Mayrhofer et al., 2018). Negative experiences with peer support groups that are not age-appropriate can have a negative impact on someone with young onset dementia, and result in reluctance to use formal dementia services (Cations et al., 2017).

Dementia symptoms can make it more difficult to travel to in-person peer support groups. Moreover, research shows that geographical and logistical challenges, such as time and money spend on travelling, are barriers to accessing (peer) support services (Cations et al., 2017; Matthias et al., 2016). Therefore, online platforms can make peer support more widely accessible. Online peer support can also be a good option for those who may not feel comfortable meeting new people in person and speak openly about their experiences with dementia. In video meetings people can still see and hear the others and feel connected. At the same time, there is the option to turn off their cameras or mute themselves, step away from the meeting for a moment, or leave the meeting at any point while being in a comfortable and safe environment.

#### Limitations and challenges of online peer support using videoconferencing platforms

This study identified that missing being together in person is one of the main limitations of online peer support. The groups that met in person or were founded with the intention of being an in-person group expressed their wish to meet in-person (again) when COVID-19 restrictions would allow that. The feeling of online support being better than no support at all was present in these groups. These findings add to previous research on the experiences of people with dementia and their families during the COVID-19 pandemic (Giebel et al., 2021). On the other hand, the online-only group whose members were from different parts of the UK and the world expressed themselves about benefits of meeting online. Other challenges included difficulties to join a meeting, having to navigate different platforms, and digital exclusion. Clear guidelines on how the platform works, having someone to help set up the necessary software, and timely reminders for the meeting can help mitigate such challenges.

### Limitations

First, this study only included people who were able to use technology and participate in video meetings. Therefore, it did not represent the views and experiences of those who are unable to use these. Second, besides one group speaking about their WhatsApp group, this study mainly focussed on real-time contact in video meetings, and did not represent how people with young onset dementia may use other platforms for online peer support, such as social media or discussion forums.

### Recommendations for practice and future research

This study identified two main reasons why people with young onset dementia experience difficulties in accessing peer support. First, there is inconsistent availability of specialised (peer) support services across the UK. Moreover, there is a lack of clear signposting to such services by healthcare professionals. This echoes previous findings by Cations et al. (2017) and Mayrhofer et al. (2018). As a result, people are often unaware that specific young onset dementia peer support exist. Second, there is still the misconception that peer support groups are mainly for older people. Better signposting to specialised young onset dementia (peer) support services is needed.

One of the challenges identified in this study is digital exclusion. Participants found that not all group members were able to take part in their online meetings. Reasons could be different levels of tech savviness and progressing dementia symptoms making it difficult to use technology. More and more of our communication is taking place online, and more and more health and social care services are being digitalised, a process that accelerated during the COVID-19 pandemic. Therefore, it is incredibly important to get more insight into the views and experiences of people with young onset dementia who do not or cannot use online (peer) support services, identify the barriers, and how to overcome these. This is important to make peer support and other services accessible to anyone living with young onset dementia who needs it.

Finally, there are many different forms of online peer support and using video meetings is only one of them. While previous research explored how people with dementia used other platforms, such as social media and discussion forums (Clare et al., 2008; Craig & Strivens, 2016; Rodriquez, 2013; Talbot et al., 2020), it remains unknown how people experience being part of such online support communities and how it affects their daily lives. Future research could focus on exploring how people with young onset dementia experience using different online platforms for peer support by using qualitative methods such as interviews or surveys.

## Conclusion

Peer support is an incredibly valuable and important source of post-diagnostic support for people with young onset dementia. However, not everyone has access to age-appropriate peer support as dementia services and support groups are often tailored towards older adults. Online platforms can make peer support more accessible for people with young onset dementia as it overcomes geographical barriers as well as barriers for those who feel uncomfortable attending an in-person group. Through video meetings people can join from the comfort of their own homes, and mute themselves or turn off their cameras at any point while still having the option to see and hear the other participants and feel part of a group. This study recommends that researchers and policy makers further explore how to implement and overcome barriers to online peer support, so that peer support is more widely accessible and signposted to people with young onset dementia.

## References

[bibr1-14713012221140468] Banbury A. Parkinson L. Gordon S. Wood D. (2019). Implementing a peer-support programme by group videoconferencing for isolated carers of people with dementia. Journal of Telemedicine and Telecare, 25(9), 572–577. DOI: 10.1177/1357633X19873793 10.1177/1357633X19873793. 31631761

[bibr2-14713012221140468] Bannon S. Reichman M. Popok P. Wagner J. Gates M. Uppal S. LeFeber L. Wong B. Dickerson B. C. Vranceanu A.-M. (2020). It together: A qualitative meta-synthesis of common and unique psychosocial stressors and adaptive coping strategies of persons with young-onset dementia and their caregivers. The Gerontologist, 62(2), e123–e139. DOI: 10.1093/geront/gnaa169 10.1093/geront/gnaa169. PMC8827330 33125490

[bibr3-14713012221140468] Barak A. Boniel-Nissim M. Suler J. (2008). Fostering empowerment in online support groups. Computers in Human Behavior, 24(5), 1867–1883. DOI: 10.1016/j.chb.2008.02.004.

[bibr4-14713012221140468] Braun V. Clarke V. (2021). Thematic analysis : A practical guide/Virginia Braun and Victoria Clarke (p. 1). SAGE.

[bibr5-14713012221140468] Carter N. Bryant-Lukosius D. DiCenso A. Blythe J. Neville A. J. (2014). The use of triangulation in qualitative research. Oncology Nursing Forum, 41(5), 545–547. DOI: 10.1188/14.ONF.545-547 10.1188/14.ONF.545-547. 25158659

[bibr6-14713012221140468] Cations M. Withall A. Horsfall R. Denham N. White F. Trollor J. Loy C. Brodaty H. Sachdev P. Gonski P. Demirkol A. Cumming R. G. Draper B. (2017). Why aren't people with young onset dementia and their supporters using formal services? Results from the INSPIRED study. PLoS ONE, 12(7), e0180935. DOI: 10.1371/journal.pone.0180935 10.1371/journal.pone.0180935. 28723931 PMC5517136

[bibr7-14713012221140468] Clare L. Rowlands J. M. Quin R. (2008). Collective strength:The impact of developing a shared social identity in early-stage dementia. Dementia, 7(1), 9–30. DOI: 10.1177/1471301207085365 10.1177/1471301207085365.

[bibr8-14713012221140468] Craig D. Strivens E. (2016). Facing the times: A young onset dementia support group: FacebookTM style. Australasian Journal on Ageing, 35(1), 48–53. DOI: 10.1111/ajag.12264 10.1111/ajag.12264. 27010874

[bibr9-14713012221140468] Dennis C.-L. (2003). Peer support within a health care context: A concept analysis. International Journal of Nursing Studies, 40(3), 321–332. DOI: 10.1016/S0020-7489(02)00092-5. 12605954

[bibr10-14713012221140468] Dugmore O. Orrell M. Spector A. (2015). Qualitative studies of psychosocial interventions for dementia: A systematic review. Aging & Mental Health, 19(11), 955–967. DOI: 10.1080/13607863.2015.1011079 10.1080/13607863.2015.1011079. 25748797

[bibr11-14713012221140468] Gerritzen E. V. McDermott O. Orrell M. (2022). Development of best practice guidance on online peer support for people with young onset dementia: Protocol for a mixed methods study. JMIR Research Protocals, 11(7), e38379. DOI: 10.2196/38379 10.2196/38379. PMC9297145 35788470

[bibr12-14713012221140468] Giebel C. Cannon J. Hanna K. Butchard S. Eley R. Gaughan A. Komuravelli A. Shenton J. Callaghan S. Tetlow H. Limbert S. Whittington R. Rogers C. Rajagopal M. Ward K. Shaw L. Corcoran R. Bennett K. Gabbay M. (2021). Impact of COVID-19 related social support service closures on people with dementia and unpaid carers: A qualitative study. Aging & Mental Health, 25(7), 1281–1288. DOI: 10.1080/13607863.2020.1822292. 32954794

[bibr13-14713012221140468] Greenwood N. Smith R. (2016). The experiences of people with young-onset dementia: A meta-ethnographic review of the qualitative literature. Maturitas, 92, 102–109. DOI: 10.1016/j.maturitas.2016.07.019. 27621246

[bibr14-14713012221140468] Hennink M. M. Kaiser B. N. Weber M. B. (2019). What influences saturation? Estimating sample sizes in focus group research. Qualitative Health Research, 29(10), 1483–1496. DOI: 10.1177/1049732318821692. 30628545 PMC6635912

[bibr15-14713012221140468] Johannessen A. Engedal K. Haugen P. K. Dourado M. C. N. Thorsen K. (2018). To be, or not to be”: Experiencing deterioration among people with young-onset dementia living alone. International Journal of Qualitative Studies on Health and Well-being, 13(1), 1490620. DOI: 10.1080/17482631.2018.1490620. 29975182 PMC6041786

[bibr16-14713012221140468] Keyes S. E. Clarke C. L. Wilkinson H. Alexjuk E. J. Wilcockson J. Robinson L. Reynolds J. McClelland S. Corner L. Cattan M. (2014). “We’re all thrown in the same boat…”: A qualitative analysis of peer support in dementia care. Dementia, 15(4), 560–577. DOI: 10.1177/1471301214529575. 24742876

[bibr17-14713012221140468] Koopmans R. Rosness T. (2014). Young onset dementia – what does the name imply? International Psychogeriatrics, 26(12), 1931–1933. DOI: 10.1017/S1041610214001574. 25382199

[bibr18-14713012221140468] Matthias M. S. Kukla M. McGuire A. B. Damush T. M. Gill N. Bair M. J. (2016). Facilitators and barriers to participation in a peer support intervention for veterans with chronic pain. The Clinical journal of pain, 32(6), 534–540. DOI: 10.1097/AJP.0000000000000297. 26340653 PMC4794408

[bibr19-14713012221140468] Mayrhofer A. Mathie E. McKeown J. Bunn F. Goodman C. (2018). Age-appropriate services for people diagnosed with young onset dementia (YOD): A systematic review. Aging & Mental Health, 22(8), 933–941. DOI: 10.1080/13607863.2017.1334038. 28621549

[bibr20-14713012221140468] McDermott O. Charlesworth G. Hogervorst E. Stoner C. Moniz-Cook E. Spector A. Csipke E. Orrell M. (2019). Psychosocial interventions for people with dementia: A synthesis of systematic reviews. Aging & Mental Health, 23(4), 393–403. DOI: 10.1080/13607863.2017.1423031. 29338323

[bibr21-14713012221140468] Millenaar J. Hvidsten L. de Vugt M. E. Engedal K. Selbæk G. Wyller T. B. Johannessen A. Haugen P. K. Bakker C. van Vliet D. Koopmans R. T. C. M. Verhey F. R. J. Kersten H. (2017). Determinants of quality of life in young onset dementia – results from a European multicenter assessment. Aging & Mental Health, 21(1), 24–30. DOI: 10.1080/13607863.2016.1232369. 27676211

[bibr22-14713012221140468] Moorhead S. A. Hazlett D. E. Harrison L. Carroll J. K. Irwin A. Hoving C. (2013). A new dimension of health care: Systematic review of the uses, benefits, and limitations of social media for health communication. J Med Internet Res, 15(4), e85. DOI: 10.2196/jmir.1933. 23615206 PMC3636326

[bibr23-14713012221140468] Oliver K. O’Malley M. Parkes J. Stamou V. La Fontaine J. Oyebode J. Carter J. (2019). Living with young onset dementia and actively shaping dementia research – the Angela Project. Dementia, 19(1), 41–48. DOI: 10.1177/1471301219876414. 31875707

[bibr24-14713012221140468] Pierse T. Keogh F. Challis D. O’Shea E. (2021). Resource allocation in dementia care: Comparing the views of people with dementia, carers and health and social care professionals under constrained and unconstrained budget scenarios. Aging & Mental Health, 26(4), 1–9. DOI: 10.1080/13607863.2021.1889969. 33663288

[bibr25-14713012221140468] Rabanal L. I. Chatwin J. Walker A. O’Sullivan M. Williamson T. (2018). Understanding the needs and experiences of people with young onset dementia: A qualitative study. BMJ Open, 8(10), e021166. DOI: 10.1136/bmjopen-2017-021166. PMC6196838 30344167

[bibr26-14713012221140468] Roach P. (2016). Young onset dementia: Negotiating future workplace roles and identities. Dementia, 16(1), 5–8. DOI: 10.1177/1471301216674420. 27758962

[bibr27-14713012221140468] Rodriquez J. (2013). Narrating dementia:self and community in an online forum. Qualitative Health Research, 23(9), 1215–1227. DOI: 10.1177/1049732313501725. 23907588

[bibr28-14713012221140468] Solomon P. (2004). Peer support/peer provided services underlying processes, benefits, and critical ingredients. Psychiatric Rehabilitation Journal, 27(4), 392–401. DOI: 10.2975/27.2004.392.401. 15222150

[bibr29-14713012221140468] Stamou V. Fontaine J. L. O’Malley M. Jones B. Gage H. Parkes J. Carter J. Oyebode J. (2021). The nature of positive post-diagnostic support as experienced by people with young onset dementia. Aging & Mental Health, 25(6), 1125–1133. DOI: 10.1080/13607863.2020.1727854. 32067481

[bibr30-14713012221140468] Stamou V. La Fontaine J. Gage H. Jones B. Williams P. O'Malley M. Parkes J. Carter J. Oyebode J. (2021). Services for people with young onset dementia: The ‘Angela’ project national UK survey of service use and satisfaction. International Journal of Geriatric Psychiatry, 36(3), 411–422. DOI: 10.1002/gps.5437. 32979287

[bibr31-14713012221140468] Talbot C. V. O'Dwyer S. T. Clare L. Heaton J. Anderson J. (2020). How people with dementia use twitter: A qualitative analysis. Computers in Human Behavior, 102, 112–119. DOI: 10.1016/j.chb.2019.08.005.

[bibr32-14713012221140468] Tong A. Sainsbury P. Craig J. (2007). Consolidated criteria for reporting qualitative research (COREQ): A 32-item checklist for interviews and focus groups. International Journal for Quality in Health Care, 19(6), 349–357. DOI: 10.1093/intqhc/mzm042. 17872937

